# Soil Effects on the Bioactivity of Hydroxycoumarins as Plant Allelochemicals

**DOI:** 10.3390/plants12061278

**Published:** 2023-03-10

**Authors:** Gracia Facenda, Miguel Real, Jose A. Galán-Pérez, Beatriz Gámiz, Rafael Celis

**Affiliations:** Instituto de Recursos Naturales y Agrobiología de Sevilla (IRNAS), CSIC, Avenida Reina Mercedes 10, 41012 Sevilla, Spain; gfacenda@irnase.csic.es (G.F.); mreal@irnase.csic.es (M.R.); jagalan@irnas.csic.es (J.A.G.-P.); rcelis@irnase.csic.es (R.C.)

**Keywords:** allelochemicals, bioherbicides, hydroxycoumarins, phytotoxicity, soil

## Abstract

Soil plays a primary role in the activity of plant allelochemicals in natural and agricultural systems. In this work, we compared the phytotoxicity of three natural hydroxycoumarins (umbelliferone, esculetin, and scopoletin) to different model plant species (*Lactuca sativa*, *Eruca sativa*, and *Hordeum vulgare*) in Petri dishes, and then selected the most phytotoxic compound (umbelliferone) to assess how its adsorption and dissipation in two distinct soils affected the expression of its phytotoxic activity. The root growth inhibitory effect of umbelliferone was significantly greater than that of esculetin and scopoletin, and the dicot species (*L. sativa* and *E. sativa*) were more sensitive to the hydroxycoumarins than the monocot species (*H. vulgare*). For all three plant species tested, the phytotoxicity of umbelliferone decreased in the following order: soilless (Petri dishes) > soil 1 > soil 2. In soil 2 (alkaline), umbelliferone displayed negligible adsorption (*K*_f_ < 0.01) and rapid biodegradation (t_1/2_ = 0.2–0.8 days), and its phytotoxicity was barely expressed. In soil 1 (acid), umbelliferone displayed enhanced adsorption (*K*_f_ = 2.94), slower biodegradation (t_1/2_ = 1.5–2.1 days), and its phytotoxicity was better expressed than in soil 2. When the microbial activity of soil 2 was suppressed by autoclaving, the phytotoxicity of umbelliferone, in the presence of soil, became similar to that observed under soilless conditions. The results illustrate how soil processes can reduce the allelopathic activity of hydroxycoumarins in natural and agricultural ecosystems, and suggest scenarios where the bioactivity of hydroxycoumarins may be better expressed.

## 1. Introduction

Coumarins represent an important class of phytochemicals that have received attention for their wide range of biological functions and potential applications as biocides [1,2]. Most natural coumarins contain an oxygenated substituent at position C7, with 7-hydroxycoumarin (umbelliferone), 6,7-hydroxycoumarin (esculetin), and 7-hydroxy-6-methoxycoumarin (scopoletin) (Supplementary Figure S1) being the most widespread in nature [2,3]. Because the hydroxyl group at C7 has been proposed to contribute to the phytotoxicity of 7-hydroxycoumarins [4], these have received attention for their possible role in plant–plant allelopathic interactions and their potential as eco-friendly herbicides [5,6,7,8,9,10]. Pan et al. [4] and Yan et al. [11] suggested that reactive oxygen species (ROS) accumulation and the inhibition of photosynthesis were involved in the phytotoxicity of umbelliferone. Graña et al. [7] indicated that the mode of action of scopoletin appeared to be analogous to that of auxinic herbicides, inducing wrong microtubule assembling, mitochondrial membrane depolarization, and ultimately cell death.

In addressing the ecological functions of plant allelochemicals and their potential applications in crop protection strategies, assessing how their activities can be altered by the presence of soil is of primary importance [12]. In spite of this, research on allelochemicals has focused on their identification and assessment of their phytotoxic potential under soilless laboratory experiments, while the role of soil has been largely overlooked [13,14,15,16,17]. Some researchers have expressed their concerns regarding the relative invalidity of these soilless laboratory bioassays, in order to explain field observations [15,18,19], stressing that addressing the role of soil is crucial to understand the complete functioning of allelochemicals in natural systems, as well as to assess the viability of their application as eco-friendly herbicides [19,20].

Disregarding the processes to which allelochemicals are subjected in the soil may result in incorrect predictions of their actual activities [21,22,23]. For example, several studies have shown that putative allelochemicals presenting great phytotoxic activity in water or artificial substrates displayed lower or no activity in natural soils [18,24,25,26]. Extensive adsorption by soil colloids and rapid degradation by soil microorganisms have been argued as the main reasons why the activity of allelochemicals is not expressed in some soils [17]. Nevertheless, it has also been pointed out that adsorption can protect allelochemicals from rapid transport and transformation losses, which may prolong their activity [10,15,27]. Therefore, the overall phytotoxic potential of allelochemicals and their performance as natural herbicides will be largely determined by the extent of their adsorption and its impact on the transformation processes that may induce a fast disappearance of the allelochemical in the soil.

The aim of this work was to get a deep insight into how adsorption and dissipation processes can influence the phytotoxic activity of hydroxycoumarins in soils, which should help for a more accurate assessment of their ecological functions and potential as eco-friendly herbicides. To achieve this goal, the following specific objectives were established: (i) to compare the phytotoxicity of the most widespread hydroxycoumarins (umbelliferone, esculetin, and scopoletin) to different model plant species (*Lactuca sativa*, *Eruca sativa*, and *Hordeum vulgare*) in Petri dish experiments, (ii) to characterize the adsorption and dissipation of the most phytotoxic compound (umbelliferone) in two soils with markedly different characteristics, and (iii) to assess the effect of the two soils on the expression of the phytotoxicity of umbelliferone compared to that observed under soilless (Petri dish) conditions.

## 2. Results

### 2.1. Petri Dish Bioassays

**Figure 1:**
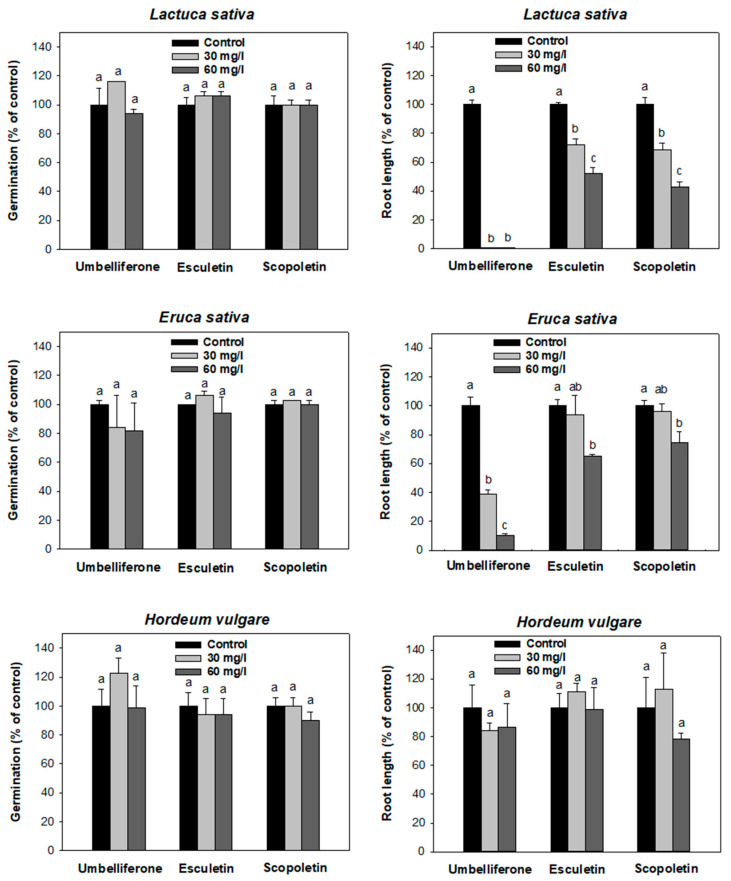
Effect of umbelliferone, esculetin, and scopoletin at concentrations of 30 and 60 mg/L on germination (**left**) and root growth (**right**) of *L. sativa*, *E. sativa*, and *H. vulgare* under Petri dish conditions (t = 6 days). Different letters above the bars indicate statistically significant differences between treatments.

The results of the Petri dish bioassays conducted to compare the effects of umbelliferone, esculetin, and scopoletin at two different concentrations (30 and 60 mg/L) on germination and seedling root growth of *L. sativa*, *E. sativa*, and *H. vulgare*, are summarized in Figure 1. None of the hydroxycoumarins affected the germination of the plant species tested, but they were found to affect the seedling root growth to different extents, following the general sequences: umbelliferone > esculetin ≈ scopoletin and *L. sativa* > *E. sativa* > *H. vulgare* (Figure 1). Thus, the root growth inhibitory activity of umbelliferone was stronger than that of esculetin or scopoletin, and the dicot species (*L. sativa* and *E. sativa*) were more sensitive to the hydroxycoumarins than the monocot species (*H. vulgare*). For the most phytotoxic hydroxycoumarin (umbelliferone), an extended dose-response study revealed *IC*_50_ values for its root growth inhibitory effect of 5.6 ± 0.2 mg/L for *L. sativa*, 11.6 ± 1.7 mg/L for *E. sativa*, and 49.9 ± 9.5 mg/L for *H. vulgare* (Supplementary Figure S2).

### 2.2. Adsorption and Dissipation of Umbelliferone in the Soils

**Figure 2:**
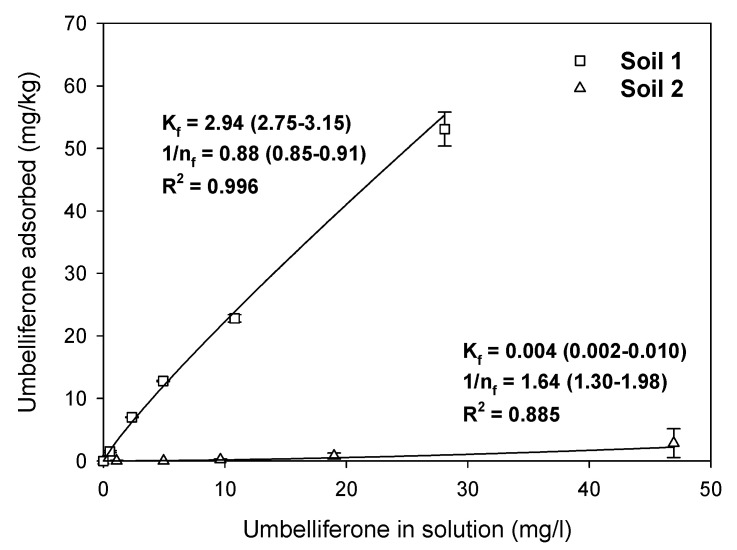
Umbelliferone adsorption isotherms on the two soils selected for the study. Symbols represent experimental data points, whereas lines are the Freundlich-fit adsorption isotherms. The Freundlich coefficients for each isotherm are indicated in the graph.

Figure 2 shows the adsorption isotherms of umbelliferone on the soils selected for this study. The rationale for selecting umbelliferone for the soil studies was that, on the basis of its greater activity (Figure 1), this compound was more likely to display allelopathic effects under real soil conditions and also greater potential for application as a natural herbicide compared to esculetin and scopoletin. Adsorption of umbelliferone on soil 2 was negligible, yielding a *K*_f_ value < 0.01 (Figure 2). At the alkaline pH of this soil (pH = 8.4, Table 1), umbelliferone (p*K*_a_ = 7.5) should have predominated as an anionic species, and organic anions are commonly repelled by the negatively charged surfaces of soil clay and organic constituents [10,28,29,30]. Adsorption was much higher in soil 1 (*K*_f_ = 2.94, Figure 2). At the acidic pH of this soil (pH = 5.8, Table 1), umbelliferone should have existed mostly as a protonated species, and the neutral forms of organic acids usually have a higher affinity for soil particles than the deprotonated (anionic) ones [29,30]. In soil 1, the adsorption isotherm was very well described by the Freundlich equation (R^2^ = 0.996), with a Freundlich coefficient of 1/*n_f_* = 0.88 ± 0.03 (Figure 2). Values of 1/*n_f_* < 1 indicate that adsorption becomes more difficult at higher solute concentrations, i.e., as adsorption sites become progressively more occupied [31].

**Figure 3:**
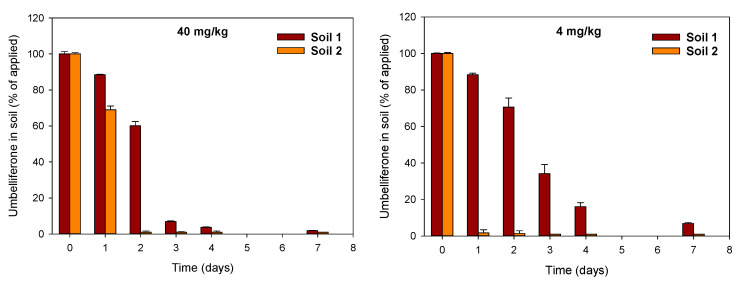
Dissipation data for umbelliferone applied at 40 and 4 mg/kg to the two soils selected for the study.

Figure 3 shows the dissipation patterns of umbelliferone in soils 1 and 2 after its application to the soils at two different initial concentrations of 40 and 4 mg/kg. Umbelliferone dissipated faster in soil 2 (t_1/2_ = 0.2–0.8 days) than in soil 1 (t_1/2_ = 1.5–2.1 days) at the two initial concentrations tested, but differences were particularly pronounced at the lower concentration of 4 mg/kg. This was because the effect of the initial concentration on the dissipation rate of umbelliferone was soil-dependent. A decrease in the application rate reduced the persistence of umbelliferone in soil 2, whereas it increased its persistence in soil 1 (Figure 3).

### 2.3. Soil Bioassays

**Figure 4:**
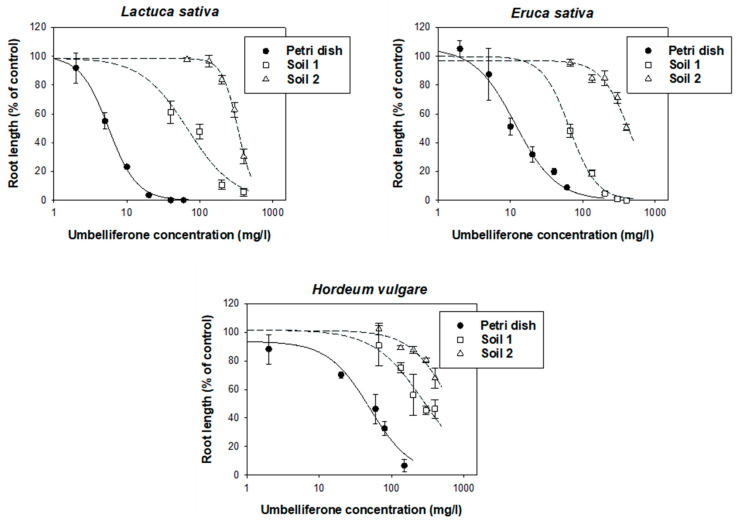
Dose-response curves for the inhibitory effect of umbelliferone on the root growth of *L. sativa*, *E. sativa*, and *H. vulgare* under Petri dish conditions and in the presence of two soils (t = 6 days).

As observed in the Petri dish study (Figure 1), the application of umbelliferone had little effect on the germination of *L. sativa*, *E. sativa*, or *H. vulgare* in soil pots (not shown); however, it led to reduced root growth and aerial biomass of all three plant species in a soil- and plant-species-dependent manner. The dose-response curves for the inhibitory effect on root growth under soil-pot conditions are compared with those obtained in Petri dishes in Figure 4, whereas the constants obtained after fitting a log-logistic 3-parameter equation (Equation (1)) to the dose-response data are compiled in Table 2. Data for the inhibitory effects of umbelliferone on the plant aerial biomass are given in Table S1 and Figure S3 of the Supplementary Material.

**Figure 5:**
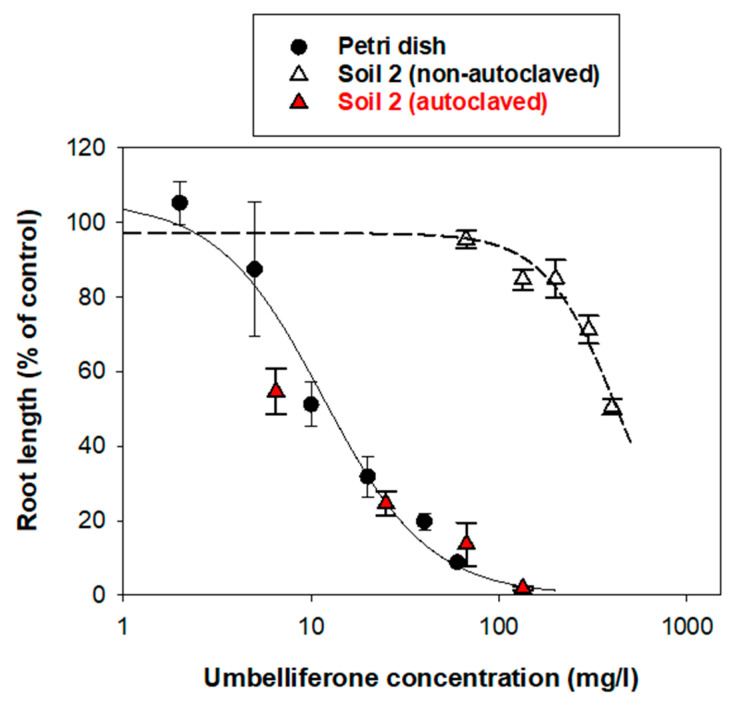
Dose-response curves for the inhibitory effect of umbelliferone on the root growth of *E. sativa* under Petri dish conditions and in the presence of non-autoclaved and autoclaved soil 2 (t = 6 days).

In the presence of the soils, the sensitivity of the plant species to umbelliferone remained as in the Petri dish study, i.e., *L. sativa* ≥ *E. sativa* > *H. vulgare*, but the root growth inhibitory activity of umbelliferone decreased notably compared to that observed in the Petri dish experiment (Figure 4). In terms of “soil factors” (Equation (4)) [24], values of 0.088–0.182 for soil 1 and 0.018–0.076 for soil 2 (Table 2) implied that the mean inhibitory concentrations of umbelliferone (*IC*_50_) became 5–10 times greater in soil 1 and 10–50 times greater in soil 2 than the values obtained in the (soilless) Petri dish experiment. For the most sensitive plant species (*L. sativa*), as an example, the *IC*_50_ shifted from 6 ± 1 mg/L in the absence of soil, to 68 ± 2 mg/L in the presence of soil 1 and 334 ± 9 mg/L in the presence of soil 2 (Table 2). Similar results were obtained when analyzing the data of aerial biomass (Supplementary Table S1). Consequently, the presence of the soils weakened the activity of umbelliferone, and this effect was particularly pronounced in soil 2. Interestingly, when the microbial activity of soil 2 was suppressed by autoclaving, the activity of umbelliferone in the soil became very similar to that observed under soilless conditions (Figure 5), pointing to a primary role of microorganisms in reducing the activity of umbelliferone in the soil.

## 3. Discussion

The higher phytotoxicity of umbelliferone compared to esculetin and scopoletin observed in the Petri dish study (Figure 1) reflected how further substitution at C6, either with a hydroxyl or methoxy group (Supplementary Figure S1), reduced the activity of 7-hydroxycoumarin. These results agree with those of a structure–activity relationship study conducted by Pan et al. [4], in which the root and shoot growth inhibitory activity of umbelliferone to pre-germinated seedlings of *L. sativa*, *S. viridis*, and *A. retroflexus* was found to be higher than that of many other 7-hydroxycoumarins tested, including esculetin.

Germination is commonly less affected by 7-hydroxycoumarins than root or shoot growth [7,10]. This is also supported by data in Figure 1, where at hydroxycoumarin concentrations of 30 and 60 mg/L, the germination of *L. sativa* and *E. sativa* was not affected, whereas root elongation already showed inhibitory effects. The finding that the monocot (*H. vulgare*) was less affected by all three hydroxycoumarins than the dicots (*L. sativa* and *E. sativa*) may be related to the fact that the mode of action of hydroxycoumarins appears to be analogous to that of auxinic herbicides [7,10], as dicots are commonly more sensitive to auxinic herbicides than monocots.

Umbelliferone displayed very distinct adsorption and dissipation behaviors in the two soils selected for this study (Figure 2 and Figure 3), showing higher adsorption and persistence in the acid soil (soil 1) than in the alkaline soil (soil 2). In a study conducted at a single initial umbelliferone concentration of 2 mg/L with eight soils, Real et al. [29] found that umbelliferone displayed greater adsorption in acid than in alkaline soils, and suggested that this was due to the fact that the molecular (uncharged) form of umbelliferone prevailing in acid soils had a higher affinity for soil particles than the ionized (anionic) form predominant in alkaline soils. This behavior was reproduced for the two soils selected for in the present study, in an extended range of initial umbelliferone concentrations of 1–50 mg/L (Figure 2).

It has been suggested that the adsorption of umbelliferone by acid soils can contribute to an increase in its persistence because adsorption makes the allelochemical less available for biological degradation, which is the principal dissipation mechanism for umbelliferone in soils [29]. In addition to this, we observed a soil-dependent effect of the initial concentration of umbelliferone on its soil dissipation rate (Figure 3). Decreasing its application dose from 40 to 4 mg/kg led to an increase in persistence in the acid soil (soil 1), but a decrease in persistence in the alkaline soil (soil 2). We believe that the different adsorption behavior of umbelliferone in the soils also accounted for this result. In soil 1, where adsorption was favored at low umbelliferone concentrations (Figure 2), the compound could have been less bioavailable, and hence more protected from biodegradation, at a low initial concentration. In soil 2, where adsorption was negligible, umbelliferone at a low concentration could have been more rapidly consumed by soil microorganisms than the allelochemical at a high concentration [33].

The root elongation inhibitory effects of umbelliferone on *L. sativa* (*IC*_50_ = 5.6 ± 0.2 mg/L), *E. sativa* (*IC*_50_ = 11.6 ± 1.7 mg/L), and *H. vulgare* (*IC*_50_ = 49.9 ± 9.5 mg/L) observed in this work under soilless conditions are in line with the phytotoxicity reported by other authors for 7-hydroxycoumarins [4,5,7,11]. It is also interesting to note that the *IC*_50_ values we obtained for the dicots are in the range of inhibitory concentrations reported for highly phytotoxic phytochemicals [34,35]. As an example, cis-abscisic acid is considered a very active natural plant growth inhibitor and its *IC*_50_ for root elongation of lettuce seedlings was reported to be 14.9 µM (3.9 mg/L) [34].

The soils selected for in this study had a remarkable effect weakening the phytotoxic activity of umbelliferone. The effect was soil-dependent, with soil 2 reducing the activity of umbelliferone to a greater extent than soil 1 (Figure 4, Table 2). Due to a lack of adsorption, as the fraction of umbelliferone immediately available for receiver plants was expected to be higher in soil 2 than in soil 1, the greater reduction in toxicity observed for soil 2 should be attributed to the more rapid biodegradation of the hydroxycoumarin in this soil, particularly at low concentrations (Figure 3). This was confirmed by the finding that, when the microbial activity of soil 2 was suppressed by autoclaving, the activity of umbelliferone in the soil became comparable to that observed under soilless conditions (Figure 5).

According to the results obtained in the present study, the phytotoxicity of umbelliferone is expected to be more weakened in alkaline than in acid soils, probably because the prolonged persistence of the compound in acid soils allows maintaining bioactive concentrations longer than in alkaline soils. In the acid soil selected for this work and taking into account the experimental conditions used in the soil-pot experiment (see Section 4.4), the umbelliferone *IC*_50_ values for root elongation of *L. sativa* and *E. sativa* (66–68 mg/L, Table 2) represented agronomic application rates of ca. 2 kg/ha. This value is comparable to the rates recommended for field application of several classes of synthetic herbicides, which is remarkable, as it is generally accepted that most known allelochemicals would need to be applied at rates higher than 10 kg/ha to achieve significant weed control [36]. Soil treatments or formulations directed to stabilize umbelliferone and increase its shelf-life in soil could further enhance the bioactivity of the compound and its potential as a natural herbicidal product.

## 4. Materials and Methods

### 4.1. Hydroxycoumarins and Soils

Umbelliferone, esculetin, and scopoletin were purchased as high-purity compounds (purity ≥ 98%) from Merck (Madrid, Spain). Umbelliferone, esculetin, and scopoletin have molecular masses of 162.1, 178.1, and 182.2, respectively, and are all weak acids, with p*K*_a1_ close to 7.5 and water solubilities near 200 mg/L. 

The two soils used in the experiments were collected from the top 0–20 cm layer of two agricultural fields located in Southern Spain (soil 1: 37°42′49″ N 06°31′35″ W, soil 2: 37°16′54″ N 06°03′59″ W). Soil samples were collected, air-dried, sieved (2 mm), and characterized at the Soil Analysis Service of IRNAS-CSIC (Table 1). The soil texture was determined by the hydrometer method, the carbonate content by the pressure calcimeter method, and the organic carbon content by dichromate oxidation. Soil pH and electrical conductivity values were measured in 1 g:2.5 mL and 1 g:5 mL soil:water slurries, respectively.

### 4.2. Petri Dish Bioassays

The phytotoxicity of umbelliferone, esculetin, and scopoletin to three model plant species (*L. sativa*, *E. sativa*, and *H. vulgare*) was compared by means of bioassays conducted in 9 cm diameter Petri dishes. Each Petri dish contained a layer of filter paper, 12 seeds of the dicots (*L. sativa* and *E. sativa*) or 9 seeds of the monocot (*H. vulgare*), provided by Vilmorin Jardin (France), and 6 mL of an individual aqueous solution of umbelliferone, esculetin, or scopoletin, at a concentration of 0 (control), 30, or 60 mg/L. The Petri dishes were sealed with Parafilm^®^ and placed in a germination chamber at 25 ± 2 °C and 16:8 h light:dark photoperiod for 6 days. For each Petri dish, the number of germinated seeds and their average radicle length were measured (t = 6 d) and expressed as a percent of the value obtained for the control. All treatments were conducted in triplicate Petri dishes.

Based on the results of the comparative phytotoxicity test, the dose-response curves for the most phytotoxic hydroxycoumarin (umbelliferone) on the three tested plant species were obtained. The experiment was conducted under conditions identical to those of the aforementioned comparative test, but using seven umbelliferone concentrations in the range of 0 to 150 mg/L. A log-logistic dose-response equation was then used to calculate the umbelliferone mean inhibitory concentration (*IC*_50_) value for the root elongation of the tested seeds:

(1) $$y=\frac{y_{0}}{1+{\left(\frac{x}{IC_{50}}\right)}^{b}}$$

where *y* and *y*_0_ are the average root lengths (% of the control) measured for the treatments at a given concentration (*x*) of umbelliferone (mg/L) and at *x* = 0, respectively, and the parameter *b* indicates the slope of the log-logistic dose-response decay curve [37].

### 4.3. Umbelliferone Adsorption and Dissipation in the Soils

The adsorption isotherms of umbelliferone on the soils selected for this study were determined by the batch equilibrium method using glass centrifuge tubes closed with Teflon^®^ caps. In triplicate, samples of 3 g of the soils were equilibrated with 8 mL of umbelliferone aqueous solutions, with initial concentrations (*C_ini_*) of 0, 1, 2, 5, 10, 20, and 50 mg/L, by shaking in an end-over-end shaker for 24 h. To avoid possible experimental artifacts caused by biodegradation losses of umbelliferone during the adsorption measurement, the soils were autoclaved 3 times at 121 °C and 200 kPa for 20 min, before initiating the adsorption experiment [29]. After the 24 h equilibration period, the tubes were centrifuged (15 min at 2000× *g*) and aliquots of the supernatant solutions were removed, filtered (0.45 µm hydrophilic PTFE syringe filters, Filter-Lab), and immediately analyzed by HPLC, to determine the concentration of umbelliferone remaining in the solution (*C_e_*). The conditions used for the analysis are detailed in Real et al. [29]. The amounts of umbelliferone adsorbed by the soils (*C_s_*) were calculated from the difference between the initial and final concentrations in solution. Tubes with the initial umbelliferone solutions without soil were also shaken for 24 h to serve as controls. Adsorption isotherms were fit to the logarithmic form of the Freundlich equation:

(2) $$\log C_{s}=\log K_{f}+\frac{1}{n_{f}}\;\log C_{e}$$

where *C_s_* (mg/kg) is the amount of umbelliferone adsorbed at the equilibrium concentration *C_e_* (mg/L), and *K_f_* (mg^1−1/nf^ l^1/n^ kg^−^^1^) and 1/*n_f_* (unitless) are the empirical Freundlich constants [38].

The persistence of umbelliferone in the soils was determined through an incubation experiment, also conducted in glass centrifuge tubes closed with Teflon^®^ caps. Samples of 1 g of the soils were placed in the tubes and treated with 0.3 mL of an aqueous solution of umbelliferone at two different concentrations (13 or 130 mg/L), to yield application rates of 4 or 40 mg/kg. For each soil and application rate tested, three replicated tubes were prepared, closed, and incubated in the dark at 25 ± 1 °C, to be removed from the incubator at t = 0, 1, 2, 3, 4, and 7 days. Immediately after being removed, the tubes were frozen for subsequent extraction with an 80:20 methanol:0.01 M H_3_PO_4_ mixture (8 mL), by shaking for 24 h in an end-over-end shaker [29]. Next, the tubes were centrifuged (15 min at 2000× *g*), and aliquots of the supernatants were filtered and analyzed by HPLC, to determine the concentration of umbelliferone remaining in the soil at each incubation time. Umbelliferone dissipation data were described using a first-order kinetic model:

(3) $$C=C_{0}\;e^{-kt}$$

where *C* (mg/kg) is the concentration of umbelliferone in soil at a time t (days), *C*_0_ (mg/kg) is the concentration at t = 0, and *k* (day^−1^) is the first-order dissipation rate constant. Half-lives were calculated as t_1/2_ = 0.693/k.

### 4.4. Soil Bioassays

The soil bioassays were conducted in plastic pots (soil surface = 20 cm^2^) containing 20 g of the soils over a layer of 30 g of sand. Triplicate soil pots were treated with 200 µL of umbelliferone solutions at different concentrations (0.4–12 g/L) prepared in acetone, followed by 6 mL of water, to give umbelliferone initial concentrations in the range of 13 to 400 mg/L (0.4–12 kg/ha), and a soil water content of 30%. The amount of acetone added to the soil evaporated rapidly and did not affect the germination or growth of the assayed plant species during the soil-pot study [10]. Controls consisted of soil pots treated identically, but without umbelliferone. Immediately after the application, 12 seeds of the dicots (*L. sativa* and *E. sativa*), or 9 seeds of the monocot (*H. vulgare*), were distributed on the soil surface and the pots were placed in the germination chamber (25 °C, 70% humidity, 16:8 h light:dark photoperiod) for 6 days. Through the course of the experiment, the soil moisture content was readjusted daily to the value of 30%. For the bioassay with *E. sativa* sown on soil 2, the experiment was repeated after autoclaving the soil, following the procedure described in Section 4.3. At t = 6 d, the number of germinated seeds, and the root length and aerial biomass of the emerged seedlings were measured and expressed as a percentage of the values obtained for the control pots. Equation (1) was used to calculate the *IC*_50_ values of umbelliferone on the root length and aerial biomass of the emerged seedlings in the presence of soil. The experimental conditions used to measure the *IC*_50_ values in the presence of soil (number of seeds, volume of water, growth chamber conditions) were comparable to those used in the Petri dish bioassays, except for the presence of soil (20 g) at a realistic soil–water content.

The “soil factor” was defined by Hiradate et al. [24] as:

(4) $$Soil\;factor=\frac{IC_{50}\;in\;the\;absence\;of\;soil}{IC_{50}\;in\;the\;presence\;of\;soil}$$

and here was calculated as an indicator of the extent to which the activity of umbelliferone was weakened by the presence of the soils. A “soil factor” ≈ 0 means that the allelochemical activity is nearly completely suppressed by the presence of soil, whereas a value = 1 means that the soil has no effect on the allelochemical activity. A hypothetical situation, where the activity of a compound could be potentiated in soil, e.g., through its transformation into more toxic compounds, would yield a soil factor > 1.

### 4.5. Data Treatment

Adsorption, dissipation, and bioassay experiments were all conducted in triplicate and standard errors were used to express variability among the replicates. Curve fits of Equations (1)–(3) to experimental data were performed using the “regression wizard” application of SigmaPlot 14.5 for Windows. Bioassay experimental data were subjected to a one-way ANOVA, followed by a post hoc Tukey’s (HSD) test for pairwise comparison between treatments.

## 5. Conclusions

In Petri dish experiments, umbelliferone, esculetin, and scopoletin inhibited the root growth of *L. sativa*, *E. sativa*, and *H. vulgare* to different extents, depending on the nature of the hydroxycoumarin and the target plant species. Substitutions at C6 in esculetin and scopoletin reduced their phytotoxicity compared to umbelliferone, and the dicot plants (*L. sativa* and *E. sativa*) were more sensitive to all three hydroxycoumarins than the monocot plant (*H. vulgare*), similarly to what was observed for auxinic herbicides.

In the presence of soils, the expression of the phytotoxicity of the most active hydroxycoumarin (umbelliferone) was significantly weakened: its *IC*_50_ values became 5 to 50 times greater than those obtained in soilless (Petri dish) experiments. The fact that microbial degradation appeared to be a primary factor weakening the phytotoxic activity of umbelliferone in the soils, points to the necessity of further studies to characterize the microbial populations and activities of soils treated with hydroxycoumarins as a key aspect determining the fate and functioning of these compounds once they reach the soil environment.

## Figures and Tables

**Table 1:** Physicochemical properties of the soils selected for this work.

Soil	Texture ^1^	Sand (%)	Silt (%)	Clay (%)	CaCO_3_ (%)	Organic C (%)	E.C. ^2^ (mS/cm)	pH
Soil 1	Sandy loam	66 ± 1 ^3^	23 ± 1	11 ± 1	0.7 ± 0.1	0.99 ± 0.01	0.053 ± 0.03	5.8 ± 0.1
Soil 2	Sandy clay loam	75 ± 1	4 ± 1	20 ± 1	0.7 ± 0.1	0.40 ± 0.03	0.062 ± 0.02	8.4 ± 0.1

^1^ According to the USDA textural triangle [32]. ^2^ Electrical conductivity. ^3^ Mean ± standard error (n = 2).

**Table 2:** Log-logistic 3-parameter constants for the effect of umbelliferone on the root length of *L. sativa*, *E. sativa*, and *H. vulgare* in bioassays conducted in the absence and the presence of soil.

	*y*_0_	*b*	*IC*_50_	R^2^	Soil Factor
	(%)		(mg/L)		
	*Lactuca sativa*				
Soilless (Petri dishes)	100 ± 2 ^1^	2.240 ± 0.125	6 ± 1	0.999	-
Soil 1	99 ± 1	1.364 ± 0.448	68 ± 2	0.964	0.088
Soil 2	99 ± 2	3.972 ± 0.488	334 ± 9	0.992	0.018
	*Eruca sativa*				
Soilless (Petri dishes)	106 ± 6	1.541 ± 0.270	12 ± 2	0.980	-
Soil 1	100 ± 3	2.333 ± 0.241	66 ± 3	0.997	0.182
Soil 2	97 ± 4	2.242 ± 0.615	435 ± 40	0.958	0.028
	*Hordeum vulgare*				
Soilless (Petri dishes)	94 ± 6	1.501 ± 0.410	50 ± 9	0.969	-
Soil 1	102 ± 6	1.243 ± 0.275	285 ± 40	0.963	0.175
Soil 2	101 ± 3	1.558 ± 0.467	656 ± 126	0.948	0.076

^1^ Value ± standard error of the calculated parameter.

## Data Availability

The data presented in this study are available on request from the corresponding author.

## Supplementary Materials

The following supporting information can be downloaded at: https://www.mdpi.com/article/10.3390/plants12061278/s1, Table S1: Log-logistic 3-parameter constants for the effect of umbelliferone on the aerial biomass of *Lactuca sativa*, *Eruca sativa*, and *Hordeum vulgare* in bioassays conducted in the presence of soil; Figure S1: Chemical structures of umbelliferone, esculetin, and scopoletin; Figure S2: Dose-response curves for the inhibitory effect of umbelliferone on the root growth of *Lactuca sativa*, *Eruca sativa*, and *Hordeum vulgare* under Petri dish conditions; Figure S3: Dose-response curves for the inhibitory effect of umbelliferone on the aerial biomass of *Lactuca sativa*, *Eruca sativa*, and *Hordeum vulgare* in the presence of two soils.
