# Supplementary material for: Soil Effects on the Bioactivity of Hydroxycoumarins as Plant Allelochemicals
Source: Plants (Basel). 2023 Mar 10;12(6):1278. doi: 10.3390/plants12061278 (PMC10053971; doi:10.3390/plants12061278)
Supplement: Supplementary file 1 [file plants-12-01278-s001.zip › plants-2256362-supplementary.pdf]

Supplementary material for

Soil effects on the bioactivity of hydroxycoumarins as plant allelochemicals

Gracia Facenda, Miguel Real, Jose A. Galán-Pérez, Beatriz Gámiz* and Rafael Celis

Instituto de Recursos Naturales y Agrobiología de Sevilla (IRNAS), CSIC, Avenida Reina Mercedes 10, 41012 Sevilla, Spain

***Corresponding Author:** B. Gámiz

Address: Instituto de Recursos Naturales y Agrobiología de Sevilla (IRNAS), CSIC

Avenida Reina Mercedes 10, 41012 Seville, Spain

E-mail: bgamiz@irnase.csic.es

Table S1. Log-logistic 3-parameter constants for the effect of umbelliferone on the aerial biomass of *Lactuca sativa, Eruca sativa,* and *Hordeum vulgare* in bioassays conducted in the presence of two soils.

	y_0 (%)	b	IC_{50} (mg/l)	R^2
		Lactuca sativa		
Soil 1	102 ± 7[a]	1.639 ± 0.349	76 ± 12	0.984
Soil 2	101 ± 3	4.026 ± 0.939	393 ± 17	0.971
		Eruca sativa		
Soil 1	102 ± 6	1.489 ± 0.258	154 ± 20	0.978
Soil 2	98 ± 1	3.925 ± 0.376	412 ± 8	0.995
		Hordeum vulgare		
Soil 1	100 ± 5	0.897 ± 0.161	169 ± 26	0.980
Soil 2	96 ± 4	6.821 ± 2.250	380 ± 16	0.943

[a] Value ± standard error of the calculated parameter.

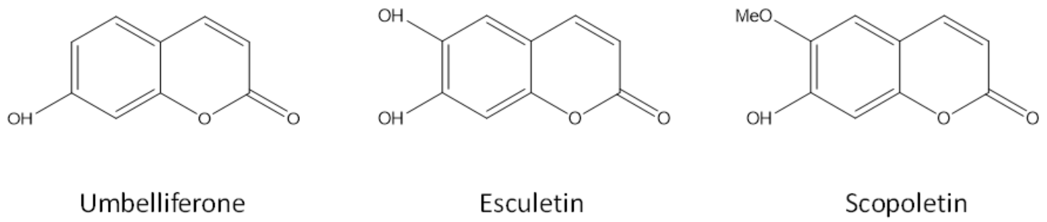

Figure S1. Chemical structures of umbelliferone, esculetin, and scopoletin.

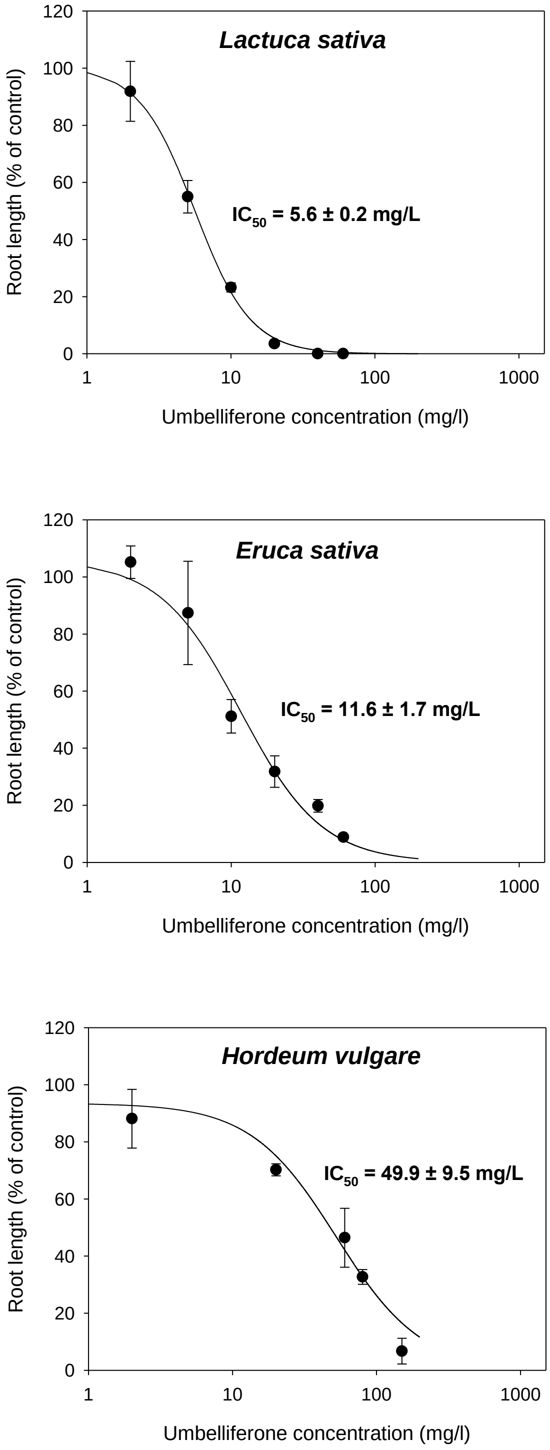

Figure S2. Dose-response curves for the inhibitory effect of umbelliferone on root growth of *Lactuca sativa*, *Eruca sativa*, and *Hordeum vulgare* under Petri-dish conditions (t = 6 days).

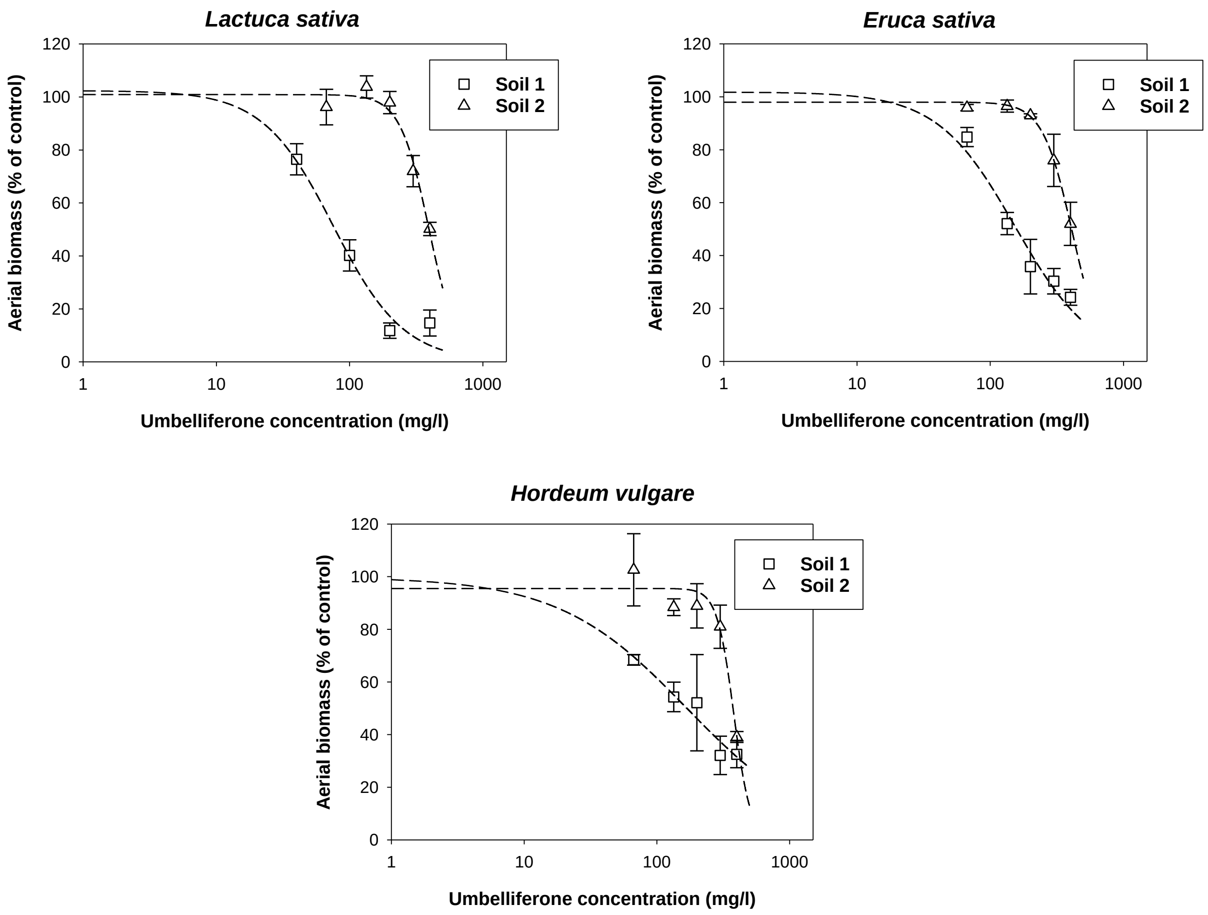

Figure S3. Dose-response curves for the inhibitory effect of umbelliferone on the aerial biomass of *Lactuca sativa*, *Eruca sativa,* and *Hordeum vulgare* in the presence of two soils (t= 6 days).
